# Genome-Wide Meta-Analysis of Systolic Blood Pressure in Children with Sickle Cell Disease

**DOI:** 10.1371/journal.pone.0074193

**Published:** 2013-09-13

**Authors:** Pallav Bhatnagar, Emily Barron-Casella, Christopher J. Bean, Jacqueline N. Milton, Clinton T. Baldwin, Martin H. Steinberg, Michael DeBaun, James F. Casella, Dan E. Arking

**Affiliations:** 1 McKusick-Nathans Institute of Genetic Medicine, Johns Hopkins University School of Medicine, Baltimore, Maryland, United States of America; 2 Department of Pediatrics, Division of Pediatric Hematology, Johns Hopkins University School of Medicine, Baltimore, Maryland, United States of America; 3 Clinical and Molecular Hemostasis Laboratory Branch, Division of Blood Disorders, National Center on Birth Defects and Developmental Disabilities, Centers for Disease Control and Prevention, Atlanta, Georgia, United States of America; 4 Department of Biostatistics, Boston University School of Public Health, Boston, Massachusetts, United States of America; 5 Department of Medicine, Boston University School of Medicine, Boston, Massachusetts, United States of America; 6 Department of Pediatrics, Vanderbilt University School of Medicine, Nashville, Tennessee, United States of America; Leibniz-Institute for Arteriosclerosis Research at the University Muenster, Germany

## Abstract

In pediatric sickle cell disease (SCD) patients, it has been reported that higher systolic blood pressure (SBP) is associated with increased risk of a silent cerebral infarction (SCI). SCI is a major cause of neurologic morbidity in children with SCD, and blood pressure is a potential modulator of clinical manifestations of SCD; however, the risk factors underlying these complications are not well characterized. The aim of this study was to identify genetic variants that influence SBP in an African American population in the setting of SCD, and explore the use of SBP as an endo-phenotype for SCI. We conducted a genome-wide meta-analysis for SBP using two SCD cohorts, as well as a candidate screen based on published SBP loci. A total of 1,617 patients were analyzed, and while no SNP reached genome-wide significance (P-value<5.0x10^-8^), a number of suggestive candidate loci were identified. The most significant SNP, rs7952106 (P-value=8.57x10^-7^), was in the *DRD2* locus on chromosome 11. In a gene-based association analysis, *MIR4301* (micro-RNA4301), which resides in an intron of *DRD2*, was the most significant gene (P-value=5.2x10^-5^). Examining 27 of the previously reported SBP associated SNPs, 4 SNPs were nominally significant. A genetic risk score was constructed to assess the aggregated genetic effect of the published SBP variants, demonstrating a significant association (P=0.05). In addition, we also assessed whether these variants are associated with SCI, validating the use of SBP as an endo-phenotype for SCI. Three SNPs were nominally associated, and only rs2357790 (*5’ CACNB2*) was significant for both SBP and SCI. None of these SNPs retained significance after Bonferroni correction. Taken together, our results suggest the importance of *DRD2* genetic variation in the modulation of SBP, and extend the aggregated importance of previously reported SNPs in the modulation of SBP in an African American cohort, more specifically in children with SCD.

## Introduction

Sickle cell disease (SCD) is an inherited hemoglobin disorder affecting approximately 1 in 600 individuals of African American ancestry in the United States [1]. The clinical manifestations of SCD begin early in life [2] and continue with an increasing incidence of adverse events, involving genetic as well as environmental factors [3]. Previous studies have demonstrated that the arterial blood pressure in steady state patients with SCD is significantly lower than that of age, sex and race matched controls [4-9]. These findings are counterintuitive in view of the well-known vascular and renal abnormalities associated with SCD and the high prevalence of hypertension in African American adults [10-12]. Recently, in SCD children with no history of overt stroke or seizures, we have reported that higher systolic blood pressure (SBP) is associated with increased risk of an silent cerebral infarction (SCI) [13], a common form of neurological injury among children with SCD, occurring in at least 27% prior to six years of life and 37% by 14 years of life [14,15]. In addition, previous clinical studies have shown that the SCD may have a deleterious effect on myocardium, which contributes to abnormal rates of change in left ventricular cavity size and systolic/diastolic function in SCD patients [16-18]. Indeed, nearly one third of adults with SCD also develop an elevated tricuspid regurgitant velocity (TRV) that is associated with a much higher death rate in SCD patients compared to patients with SCD without elevated TRV. About 5-10% of these patients have true pulmonary hypertension [19]. Identifying genetic factors associated with systolic blood pressure (SBP) may help define both patho-physiological mechanisms, as well as identify patients at increased risk for SCI.

Studies of familial aggregation provide significant evidence that blood pressure is a highly heritable trait [20]; however, these estimates provide no information as to whether the same genetic variants influence blood pressure across human populations. In 2009, two genome-wide association studies (GWAS) and meta-analysis of inter-individual blood pressure variation in adults were conducted by the Cohorts for Heart and Aging Research in Genome Epidemiology (CHARGE) Consortium [21] and the Global Blood Pressure Genetics (Global BPgen) Consortium [22], leading to the identification of a number of genomic loci implicated with these traits, including 7 for SBP. Subsequently, these consortia were combined and expanded to form the International Consortium for Blood Pressure (ICBP), who reported many additional novel loci for these traits utilizing individuals of European (N=200,000), East Asian (N=30,000), South Asian (N=24,000) and African (N=20,000) descents [23]. In the present study, we sought to apply two complementary approaches for identifying SBP variants in individuals with SCD. First, we performed a genome-wide association study for SBP in SCD cohorts of African American ancestry. Second, we attempted to validate the ICBP identified SBP loci in these patients. Finally, we assessed whether these reported variants are also associated with SCI, exploring the use of SBP as an endo-phenotype for SCI.

## Methods

### Study and Population Samples

This study includes two unrelated admixed African American ancestry SCD cohorts. Study protocols of both cohorts were approved by the Institutional Review Board (IRB) of Johns Hopkins University and Boston Medical Center. Additionally, IRB approval was acquired from all of the participating sites for subject enrollment and conducted in accordance with institutional guidelines.

### Silent Infarct Transfusion (SIT) Trial cohort

The Silent Infarct Transfusion (SIT) Trial is an international, multi-center clinical study funded by the National Institute of Neurological Disorders and Stroke (NINDS) (http://sitstudy.wustl.edu/) [24]. All the participants included in our study are of African American ancestry and written informed consent was obtained from parents of the SCD-affected individuals. For each patient, DNA was collected from Epstein-Barr virus (EBV) transformed lymphoblasts using Puregene Genomic DNA Purification kits (Gentra Systems, Inc). Demographic and phenotypic information were collected for each participant and the inclusion criteria for the recruitment were age (5-15 years) and hemoglobinopathy diagnosis (either Hb SS or Hb SB^0^-thalassemia). Details of the SIT Trial study design are given elsewhere [24].

### Cooperative Study of Sickle Cell Disease

The Cooperative Study of Sickle Cell Disease (CSSCD) was a multi-institutional prospective longitudinal study of SCD funded by the National Heart, Lung, and Blood Institute (NHLBI) of the National Institutes of Health (NIH) [25]. In our study, we only included CSSCD participants who are of African American ancestry, and to match the samples with the SIT Trial cohort, age inclusion criteria of <15 years was used. Details of the CSSCD study design are given elsewhere [25].

### Phenotype Assessment

#### Systolic Blood Pressure (SBP)

In the SIT Trial, a single measurement of blood pressure was obtained at well-visit for children with SCD. No guidelines were formulated to include uniform assessment. In the CSSCD cohort, longitudinal blood pressure measurements were collected during routine visits but not during episodes of acute illness. Blood pressure measurements were made by study nurses or physicians following the procedure described in the study manual of operations [25]. Subjects were asked not to smoke for at least 30 minutes prior to the examination and were allowed to rest quietly for at least 5 minutes before the measurement was made. A single measurement of pressure was made with the patient in the sitting position. Mercury sphygmomanometers were used for the measurement with a cuff size sufficient to cover two thirds of the upper arm. Systolic and diastolic pressures were reported as the first and fifth Korotkoff sounds, respectively.

#### Silent Cerebral Infarction (SCI)

A magnetic resonance imaging (MRI) was obtained from all participants and the presence or absence of SCI was adjudicated by a blinded panel of three expert neuro-radiologists.

### Genotyping and Quality Control

Genotyping of the SIT Trial cohort was performed in two stages. For stage 1, a subset of 573 samples, along with 24 International HapMap Consortium [26] controls and 13 known duplicates, were genotyped at the Center for Inherited Disease Research (CIDR) at Johns Hopkins University using the Illumina HumanHap650Y SNP array (Illumina Inc., San Diego CA, USA). This array contains approximately 661,000 SNPs, of which ~100,000 were selected as tags for populations with African ancestry [27]. The Beadstudio software (Illumina Inc.) was used to cluster the data and samples with <96.5% call rate were re-genotyped. The reproducibility, calculated from duplicate pairs was 99.98% and genotype concordance with HapMap data was 99.76%. In stage 2, 509 samples were genotyped at the Center for Disease Control (CDC) at Washington University using the Illumina Infinium HumanOmni1-Quad SNP array (Illumina Inc.) and achieved the call rate of ≥96%.

For quality control (QC), we performed several rounds of data cleaning and a 96.5% cutoff was used for the sample call rate and SNP coverage in the combined SIT Trial data (n=1082), and resulted the exclusion of 6 individuals and 1,260 SNPs from the study. Cryptic relatedness was determined by examining pair-wise identity-by-descent (IBD), and 77 samples were identified as first-degree relatives and dropped from the study. Given the admixed nature of the study participants, we used principal component analysis (PCA) as implemented in EIGENSTRAT [28] to both identify genetic outliers (>6 standard deviations on any of the top ten principal components) and correct for any potential residual population substructure. In the SIT Trial, twenty-six individuals were identified as genetic outliers and further excluded from the analysis. Additionally, 48 samples, due to incomplete phenotype data, were also dropped from the study, leaving 925 samples (males: 51.9% & females: 48.1%) for the subsequent GWAS analysis. Among them, 89% of the samples (n=826/924) had a confirmed SCI status and 98 individuals (11%) were not classified. In total, 251 SCI positive and 575 SCI negative samples were assigned as cases and controls, respectively.

In the CSSCD cohort, DNA samples were genotyped at Boston University using Illumina Human610-Quad SNP arrays (Illumina, San Diego, CA, USA) with approximately ~600,000 SNPs. All samples were processed according the manufacturer’s protocol and the BeadStudio software was used to make genotype calls utilizing the Illumina pre-defined clusters. Samples with <95% call rate were removed and SNPs with a call rate <97.5% were re-clustered. After re-clustering, SNPs with call rates >97.5%, cluster separation score >0.25, and excess heterozygosity between -0.10 and 0.10 were retained in the analysis. The pair-wise IBD was used to identify cryptic relatedness and PCA was applied to detect genetic outliers. After excluding these samples and following the SIT Trial age inclusion criteria (<15 years), analysis was restricted to a dataset of 692 samples (males: 52% & females: 48%).

### Merging GWAS Data and Imputation

To infer un-genotyped SNPs and fill-in missing data in the SIT Trial and CSSCD cohort, HumanHap650Y, HumanOmni1-Quad and Human610-Quad SNP array datasets were merged and subsequently imputation was performed for autosomes using a Hidden Markov model, as implemented in the MaCH software [29] (version 1.16) (http://www.sph.umich.edu/csg/abecasis/MaCH/), with 50 rounds and 200 states. QC was performed both before and after imputation and poorly imputed SNPs (RSq <0.5, squared correlation between imputed and true genotypes) were excluded and total 1,019,297 SNPs were analyzed.

### SNP Selection for Validation

Due to the lack of any published studies that report genetic determinants for SBP in children, and/or more specifically in African American children at genome-wide significance, we used the ICBP identified SBP SNPs for validation. To validate results from the ICBP study in SCD patients, we examined the 28 SNPs that were reported associated with SBP [23]. For SNPs that were not available on our genotyping array, a close proxy for the index SNP with criteria of r^2^ ≥ 0.6 from HapMap Phase III (release 2, ASW panel) [30] or 1000 Genomes project (Pilot 1, YRI panel) [31] was used.

### GWAS Meta-analysis and Statistics

All quality control measures in both cohorts were performed using the PLINK software package [32], version 1.06 (http://pngu.mgh.harvard.edu/purcell/plink/). In the SIT Trial cohort, to account for the uncertainty of the imputed data, the estimated allele dosage was analyzed using ProABEL [33] under a multivariate linear regression framework. Association for each SNP was assessed after adjusting for age, sex, height and the 1^st^ principal component, and assuming an additive effect of allele dosage on SBP. In the CSSCD cohort, SBP measurements were available for longitudinal time points and data was analyzed using a linear mixed effect model using the lme4 package (http://lme4.r-forge.r-project.org/) in R (http://www.r-project.org/) (version 2.14.1). In the mixed effect model, age, sex, height and the 1^st^ principal component were used as fixed effect covariates, while multiple SBP measurements within each individual were treated as random effect. GWAS results from both the cohorts were meta-analyzed using inverse-variance weighted fixed-effect models as implemented in METAL (http://www.sph.umich.edu/csg/abecasis/metal) [34]. The variance inflation factor for genomic control (λ_GC_), as described by Devlin and Roeder [35], was evaluated in each cohort prior to meta-analysis and a total of 1,019,297 SNPs were meta-analyzed. To explore the previously published SBP associated SNPs [23], a one-sided test of significance was used. To estimate the effect of these SNPs on SCI (data available only from the SIT Trial cohort), multivariate logistic regression was used after adjustments for age, sex and 1^st^ principal component. The genetic risk scores for SBP and SCI were constructed (based on previously published SBP variants and weighted according to their effect sizes) using an R package Genetics ToolboX (http://cran.r-project.org/web/packages/gtx/index.html). To construct the genetic risk scores, this R package uses the same underlying statistics which was used by the ICBP [23] and can be defined as follows: Assuming a set of *m* SNPs from a discovery panel, for the *i*-th SNP in the *j*-th individual denotes *x*_*ij*_ as the coded genotype (for directly genotyped SNPs) or estimated allele dosage (in case of imputation). If the set of regression coefficients of the reported SNPs are *w*_*1*_, *w*_*2*_. . *w*_*m*_, then the risk score for individual *j* is defined as: *s*_*j =*_ *s*_*o*_ + *w*_*1*_ *x*_*1j +*_ *w*_*2*_ *x*_*2j + … +*_ *w*_*m*_ *x*_*mj*_, where *s*_*o*_ is the intercept. In our analyses, we specify the coefficient *w*_*1*_, *w*_*2*_. . *w*_*m*_, to be the effect sizes (in mmHg per coded allele). Further, to identify known functional regulatory variants within or in proximity to the loci of interest, the GTEx (Genotype-Tissue Expression) expression quantitative trait loci (eQTL) database was queried (http://www.ncbi.nlm.nih.gov/gtex/GTEX2/gtex.cgi) [36].

### Gene-based Association Testing

To increase power by combining independent associations within a gene into a single, stronger aggregated signal, gene-based association tests were performed using GWiS [37]. GWiS uses greedy Bayesian model selection (selecting a minimal subset of associated SNPs within a gene) to identify independent effects and estimates overall significance through permutation. For each test statistics, using meta-analysis summary data, the gene P-values were computed using 1,000,000 permutations and utilizing the 1000 Genomes Project ASW panel as a reference population to account for linkage disequilibrium (LD) between SNPs.

## Results

### Genome-wide single SNP association

**Figure 1:**
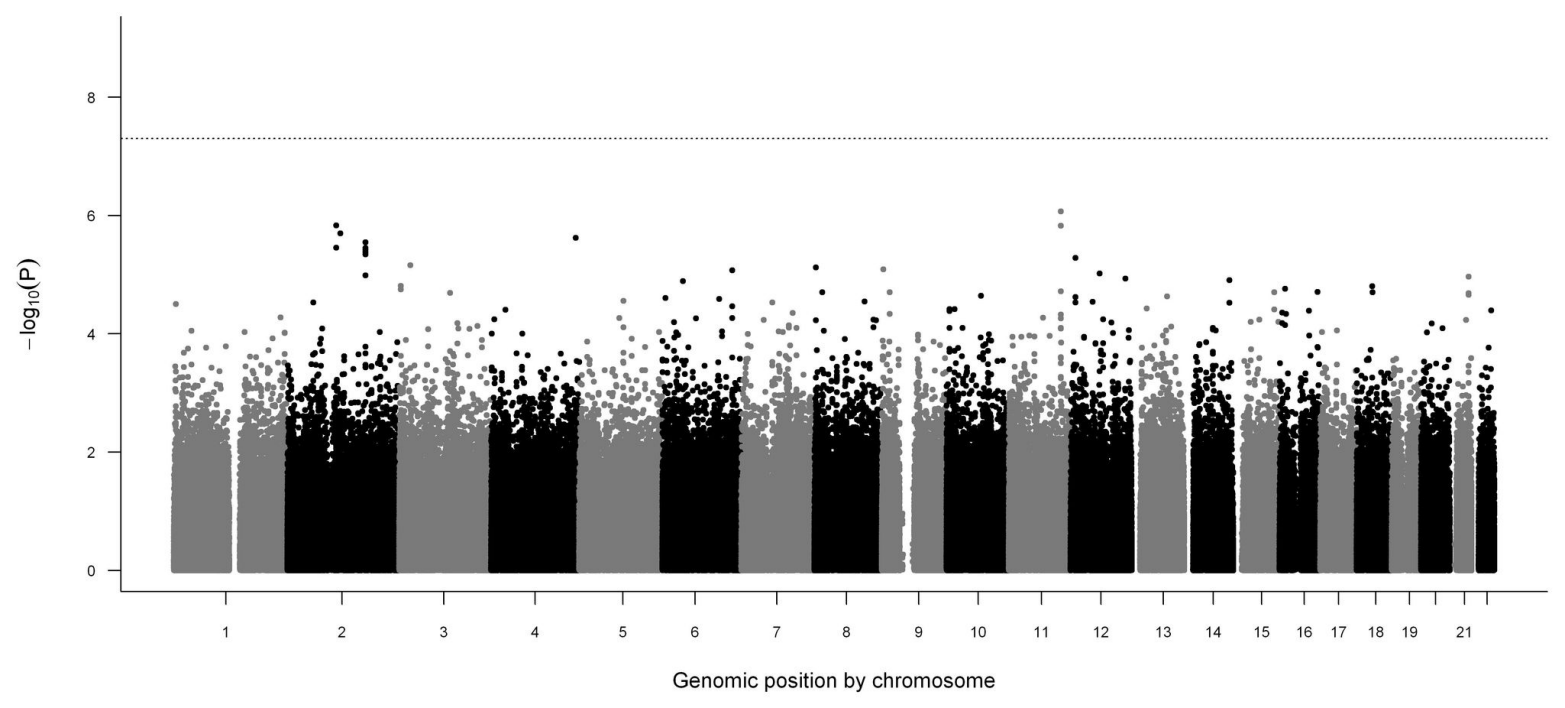
Manhattan plot showing the association of SNPs with systolic blood pressure (SBP). The genome-wide distribution of -log_10_ P-values are plotted against the physical position of each SNP on each chromosome. The threshold for genome-wide significance (P-value < 5.0x10^-8^) is indicated by the horizontal dashed line.

Genome-wide association and meta-analysis was performed for SBP in 1,617 subjects (843 males, 774 females) from the SIT Trial and CSSCD cohorts. The average ages of studied samples from the SIT Trial and CSSCD cohorts were 8.96 and 9.57 years, respectively. Detailed demographic and clinical characteristics for the study subjects are described in Table 1. The observed P-values show no early departure from the null (Figure S1), indicating minimal inflation (λ_GC_=0.998) in test statistics due to potential population stratification and/or cryptic relatedness. None of the SNPs reached genome-wide significance (P-value <5.0x10^-8^) (Figure 1). However, a number of suggestive candidate loci (P-value <5.0x10^-5^) were identified that approached genome-wide significance (Table 2). The most significant signal was observed for rs7952106 (P-value: SIT Trial=6.40x10^-3^; CSSCD=3.94x10^-5^; Meta-analysis=8.57x10^-7^). This SNP is located ~78 kb 5’ upstream of the dopamine receptor D2 subtype (*DRD2*) gene on chromosome 11. rs7952106 is a common SNP with a minor allele frequency (MAF) of 23%, and directly genotyped in both the cohorts. The direction effect of the minor allele (G) is consistent across both GWAS (SIT Trial: Effect size=1.65 mmHg/allele; CSSCD: Effect size=1.50 mmHg/allele) and associated with increase in SBP. A second DRD2 intronic SNP (rs17529477; minor allele frequency [A] =12%) showed the same direction effect (Effect size=1.76 mmHg/minor allele; P-value=1.93x10^-5^) and was in low LD with rs7952106 (r^2^: SIT Trial=0.25 and CSSCD=0.24).

**Table 1:** Demographic and clinical characteristics of the SIT Trial and CSSCD cohorts.

**Variables**	**SIT Trial**	**CSSCD**	P-value*
Sex, n (%)			
Men	483 (52.2%)	360 (52%)	0.89
Age (in years), mean ± SD	8.96 ± 2.44	9.57 ± 2.91	0.002
Baseline SBP^†^ (mm Hg), mean ± SD	108.2 ± 11.51	100.8 ± 10.99	<0.001
Baseline DBP^‡^ (mm Hg), mean ± SD	60.58 ± 8.06	59.21 ± 10.27	0.04
Height (cm), mean ± SD	128.70 ± 14.37	131.5 ± 16.08	0.008
Hemoglobin (g/dl), mean ± SD	8.11 ± 1.08	8.13 ± 0.96	0.41
Hematocrit (%), mean ± SD	23.34 ± 3.42	23.57 ± 3.10	0.12
White blood counts (Cu), mean ± SD	12.58 ± 5.25	12.01 ± 2.56	0.13
Fetal hemoglobin (%), mean ± SD	8.93 ± 5.75	8.00 ± 7.45	0.014

The shown laboratory assessments for both the cohorts are at baseline

* P-values for continuous and categorical variable comparisons were generated using wilcoxon rank sum and Kruskal-Wallis test, respectively

† SBP indicates systolic blood pressure

‡ DBP indicates diastolic blood pressure

**Table 2:** Association summary of top scoring independent loci of systolic blood pressure GWAS.

**Chr**	**SNP**	**Position**	**Nearest Gene**	**Location**	**CA/OA**	**CAF**	RSq^*^	**SIT Trial^†^ (n=925, age <15 yrs)**				**CSSCD^‡^ (n=692, age <15 yrs)**				**Meta-analysis**			
								**Effect^†^**	**SE**	**P-value**		**Effect^‡^**	**SE**	**P-value**		**Effect^§^**	**SE**	**P-value**	**Direction**
2	rs1669539	105,981,706	*NCK2- C2orf40*	Intergenic	T/C	0.74	0.92	1.31	0.62	0.036		1.6	0.37	1.29x10^-5^		1.52	0.32	1.47x10^-6^	++
2	rs3105491	114,697,437	*ACTR3- DPP10*	Intergenic	T/G	0.3	0.76	0.61	0.78	0.43		1.71	0.35	1.08x10^-6^		1.52	0.32	2.0x10^-6^	++
2	rs11568377	169,561,466	*ABCB11*	Coding Synonymous	A/G	0.17	1	-0.81	0.68	0.23		-1.95	0.41	2.41x10^-6^		-1.64	0.35	3.54x10^-6^	−
3	rs17586876	2,939,182	*CNTN4*	Intron	A/G	0.92	0.92	0.58	1.13	0.61		-2.95	0.58	3.02x10^-7^		-2.22	0.51	1.56x10^-5^	+-
3	rs826221	24,243,440	*THRB*	Intron	A/G	0.93	0.87	-1.9	1.03	0.06		-3.03	0.72	2.61x10^-5^		-2.66	0.59	6.96x10^-6^	−
4	rs10520528	183,497,595	*ODZ3*	Intron	T/G	0.43	1	1.63	0.52	0.0018		1.17	0.32	0.0003		1.3	0.27	2.40x10^-6^	++
6	rs7769148	44,412,354	*AARS2- SPATS1*	Intergenic	A/G	0.23	0.63	-2.43	0.68	0.0004		-1.51	0.56	0.0066		-1.88	0.43	1.30x10^-5^	−
6	rs4869931	151,051,582	*PLEKHG1*	Intron	A/G	0.47	0.97	0.12	0.53	0.81		1.61	0.32	3.90x10^-7^		1.21	0.27	8.48x10^-6^	++
8	rs1442407	3,078,991	*CSMD1*	Intron	T/G	0.1	1	1.45	0.87	0.1		2.16	0.51	2.33x10^-5^		1.98	0.44	7.58x10^-6^	++
9	rs7045640	2,968,377	*KIAA0020- RFX3*	Intergenic	A/C	0.76	0.76	-2.18	0.6	0.0003		-1.43	0.52	0.0056		-1.75	0.39	8.13x10^-6^	−
11	rs17529477	112,822,277	*DRD2*	Intron	A/G	0.12	1	1.79	0.81	0.027		1.75	0.48	0.00024		1.76	0.41	1.93x10^-5^	++
**11**	**rs7952106**	**112,929,768**	***DRD2- MIR4301***	**5'-Upstream**	**G/T**	**0.23**	**1**	**1.65**	**0.61**	**0.0064**		**1.5**	**0.36**	**3.94x10^-5^**		**1.54**	**0.31**	**8.57x10^-7^**	**++**
12	rs11053548	10,061,994	*CLEC12B*	Intron	A/G	0.91	1	2.77	0.92	0.0022		1.88	0.54	0.00049		2.12	0.46	5.19x10^-6^	++
12	rs1989584	62,619,479	*SRGAP1*	Intron	T/G	0.36	1	-1.14	0.53	0.034		-1.27	0.33	9.68x10^-5^		-1.23	0.28	9.64x10^-6^	−
12	rs11064853	118,544,491	*LOC387890*	Intron	A/G	0.07	0.67	2.37	1.14	0.038		3.54	0.9	7.75x10^-5^		3.09	0.71	1.17x10^-5^	++
14	rs9743626	97,844,745	*C14orf64- C14orf177*	Intergenic	A/G	0.1	1	2.37	0.86	0.0063		1.82	0.53	0.00056		1.97	0.45	1.24x10^-5^	++
16	rs289673	12,338,371	*SNX29*	Intron	A/G	0.67	0.68	-1.63	0.61	0.0077		-1.53	0.45	0.00073		-1.57	0.36	1.75x10^-5^	−
16	rs7499397	82,177,970	*CDH13*	Intron	T/C	0.38	0.92	-1.8	0.57	0.0017		-1	0.32	0.0017		-1.19	0.28	1.98x10^-5^	−
18	rs1543073	34,149,356	*CELF4*	5' Upstream	A/G	0.41	0.93	-0.52	0.56	0.36		-1.44	0.32	7.97x10^-6^		-1.21	0.28	1.57x10^-5^	−
21	rs2210268	40,685,128	*DSCAM*	Intron	T/G	0.83	1	-1.8	0.66	0.0062		-1.43	0.41	0.00049		-1.54	0.35	1.08x10^-5^	−

Chr: chromosome; CA: coded allele; OA: other allele; CAF: coded allele frequency; RSq: Imputation quality (squared correlation between imputed and true genotypes)

Genomic positions are in reference to NCBI build 36.3

Table includes SNPs with independent effects and whose significance in the meta-analysis is <5.0x10^-5^.

* Directly genotyped SNPs are marked as 1

† Effect size is based on multivariate linear regression (adjusted for age, sex, height and 1st principal component)

‡ Effect size is based on linear mixed model (age, sex, height, 1st principal component and the index SNP were treated as fixed effect covariates and longitudinal measurements of systolic blood pressure of each individual was used as a random effect)

§ Effect size is based on inverse-variance weighted fixed-effect meta-analysis

The effect size estimates corresponds to mmHg per coded allele for SBP

### Gene-based association analysis

Given the suggestion of multiple independent signals in *DRD2*, we performed a gene-based test combining independent associations within a gene (using 20kb flanking region) and obtained a P-value for each gene using permutation. In total, 32,155 autosomal genes were tested, and results are shown in Table **3**. Among all the tested genes, none met the genome-wide significant criteria (P-value < 2.0x10^-6^). The most significant gene was *MIR4301* (micro-RNA4301, Gene ID: 100422855) with a P-value=5.2x10^-5^ (Table **3**). *MIR4301* is a 65 base-pair long non-coding RNA at chromosome 11 and contained within the *DRD2* intronic region, and shares the same set of associated SNPs as observed for the *DRD2* gene. To examine micro-RNA target binding predictions, we used the software RNA22 (version 1.0) (http://cbcsrv.watson.ibm.com/rna22.html) [38]. *MIR4301* showed a predicted target site in the 3’ UTR region of the *DRD2* transcript (ENSEMBL: ENST00000355319). In our study, the observed suggestive genetic signals of *DRD2* region SNPs and the prediction of *MIR4301* binding to *DRD2* as a potential target, suggests the plausible involvement of *DRD2* region in the regulation of SBP in SCD cohorts. Examining the GTEx database, which queries lymphoblastoid, liver, brain cerebellum, frontal cortex, and temporal cortex tissue, we found no known eQTLs within or in close proximity of this locus.

**Table 3:** Gene-based association analysis of systolic blood pressure.

**Chr**	**Gene**			**SNPs^†^**	**Tests^‡^**	**K^§^**	P-value*
	**Name**	**Start**	**End**				
1	*LOC400750*	39,154,602	39,196,561	13	6.31	1	0.0011
2	*ABCB11*	169,759,449	169,907,833	172	22.99	1	3.9x10^-4^
4	*MRFAP1*	6,622,445	6,664,449	14	7.59	1	7.4x10^-4^
5	*MIR583*	95,394,842	95,434,916	14	4.77	1	1.5x10^-4^
6	*LOC134997*	24,956,605	24,997,415	29	7.30	1	7.1x10^-4^
6	*SPATS1*	44,290,397	44,364,904	38	11.54	1	4.1x10^-4^
6	*PLEKHG1*	150,940,999	151,184,799	141	29.94	1	0.0011
8	*LOC100507422*	48,484,706	48,525,170	3	2.95	1	9.0x10^-4^
9	*CARM1P1*	2,923,562	3,073,404	89	17.04	1	3.1x10^-4^
10	*NUDT13*	74,850,210	74,911,581	10	4.55	1	3.1x10^-4^
10	*USP54*	75,237,296	75,355,433	10	3.59	1	2.6x10^-4^
11	*OR5D15P*	55,534,441	55,575,382	13	7.34	1	0.001
11	*OR5W1P*	55,650,819	55,691,621	31	8.51	2	3.3x10^-4^
11	*DRD2*	113,260,317	113,366,001	61	14.46	2	0.0011
11	*MIR4301*	113,300,745	113,340,810	17	7.13	2	5.2x10^-5^
12	*CLEC1B*	10,125,660	10,171,899	49	9.47	1	2.0x10^-4^
12	*PRKAG1*	49,376,055	49,432,629	10	3.69	1	2.9x10^-4^
12	*SRGAP1*	64,218,541	64,561,613	101	25.84	1	9.3x10^-4^
12	*TMEM233*	120,011,264	120,099,363	24	8.01	2	1.4x10^-4^
13	*KCTD12*	77,434,304	77,480,540	23	10.78	1	6.7x10^-4^
15	*BLM*	91,240,579	91,378,686	41	11.18	1	9.5x10^-4^
19	*MIR4746*	4,425,975	4,466,045	13	2.70	1	0.0010
20	*CYB5AP4*	22,846,128	22,886,676	13	6.16	1	6.9x10^-4^
21	*DSCAM-AS1*	41,735,010	41,777,285	23	9.36	1	1.8x10^-4^

Gene start and end positions includes ±20 Kb of 5' and 3'-untranslated regions of the genes.

The threshold for genome-wide gene significance (2.0x10^-6^) was established using permutation.

† Number of SNPs tested in the gene

‡ Effective number of SNPs in the gene

§ Number of SNPs in the model

Significance for the gene model

### Association of previously reported SBP loci

A total of 29 independent chromosomal loci associated with blood pressure have been reported from the ICBP meta-analysis, of which 28 show strong evidence for association with SBP. We attempted to validate these loci in the combined SCD cohorts. To ensure uniform comparison of the genetic effect and its direction, the SNPs were analyzed according to the reported coded alleles (under an additive genetic model). We were able to test 27 of these SNPs directly or with a proxy SNP (r^2^≥0.6), and 15/27 SNPs showed the same direction affect on SBP as reported in the ICBP study (Table **4**, Figure **S2**). Of the directly genotyped/imputed or proxy SNPs, 4 were nominally significant in the combined SCD cohorts, and 6 were significant at P-value <0.10 (Table **4**), demonstrating a clear enrichment of signal (P-value =0.0045). However, none of the 27 tested SNPs was significant after Bonferroni correction (α=0.05/27=1.85x10^-3^). Given the limited power to detect significance for individual SNPs in the relatively smaller SCD samples (Figure **S3**), we constructed a genetic risk score for SBP incorporating the 27 previously reported SNPs; weighted according to effect sizes observed in the ICBP meta-analysis. The risk score derived from these 27 directly genotyped or proxy SNPs was nominally associated with SBP (P-value= 0.05), demonstrating the role of these SNPs in aggregate in the modulation of SBP in the SCD cohort.

**Table 4:** Association of previously reported SBP-associated SNPs with SBP and SCI.

**Chr**	**Genes**	**SNP (Position)**	**ICBP GWAS**				**Genotyped or Imputed data available in both cohorts (Proxy SNP)**	**Systolic Blood Pressure (SIT Trial + CSSCD cohort) (n=1,617)**					**Silent Cerebral Infarction (SIT Trial cohort) (n=826)**		
			**CA/OA**	**CAF**	**Reported effect of CA on SBP risk**	**ReportedP-value**		**CA/OA**	**CAF**	**Observed effect of CA on SBP risk^†^**	Observed P-value*		**OR^‡^**	**95% CI**	**P-value**
1	*MTHFR-NPPB*	rs17367504 (11,785,365)	G/A	0.15	Decreases	8.72x10^-22^	Yes	G/A	0.1	Decreases	0.37		1.06	(0.74-1.53)	0.75
1	*MOV10*	rs2932538 (113,018,066)	G/A	0.75	Increases	1.17x10^-9^	Yes	G/A	0.83	Decreases	NA		1.33	(0.87-2.02)	0.18
3	*SLC4A7*	rs13082711 (27,512,913)	T/C	0.78	Decreases	1.51x10^-6^	No (rs3755652) (27,447,940) (r^2^=0.71)	C/T	0.93	Increases	NA		0.86	(0.55-1.35)	0.51
3	*MECOM*	rs419076 (170,583,580)	T/C	0.47	Increases	1.78x10^-13^	No (rs16853620) (170,582,811) (r^2^=0.97)	A/G	0.41	Increases	0.42		0.85	(0.69-1.05)	0.13
4	*FGF5*	rs1458038 (81,383,747)	T/C	0.29	Increases	1.47x10^-23^	Yes	T/C	0.08	Decreases	NA		1.25	(0.84-1.85)	0.27
4	*SLC39A8*	rs13107325 (103,407,732)	T/C	0.05	Decreases	3.27x10^-14^	Yes	T/C	0.01	Increases	NA		0.62	(0.27-1.39)	0.24
4	*GUCY1A3-GUCY1B3*	rs13139571 (156,864,963)	C/A	0.76	Increases	1.16x10^-6^	No Proxy Found	-	-		-		-	-	-
5	*NPR3-C5orf23*	rs1173771 (32,850,785)	G/A	0.6	Increases	1.79x10^-16^	Yes	G/A	0.78	Decreases	NA		0.91	(0.70-1.18)	0.48
5	*EBF1*	rs11953630 (157,777,980)	T/C	0.37	Decreases	3.02x10^-11^	No (rs12187017) (157,781,873) (r^2^=0.92)	A/G	0.19	Decreases	0.31		1.11	(0.76-1.62)	0.59
6	*HFE*	rs1799945 (26,199,158)	G/C	0.14	Increases	7.69x10^-12^	Yes	G/C	0.02	Decreases	NA		1.27	(0.38-4.22)	0.7
6	*BAT2-BAT5*	rs805303 (31,724,345)	G/A	0.61	Increases	1.49x10^-11^	Yes	G/A	0.39	Increases	0.12		1.13	(0.91-1.42)	0.27
10	*CACNB2(5')*	rs4373814 (18,459,978)	G/C	0.55	Decreases	4.81x10^-11^	No (rs2357790) (18,463,386) (r^2^=0.93)	C/T	0.55	Decreases	0.05		0.75	(0.57-0.99)	0.04
10	*CACNB2(3')*	rs1813353 (18,747,454)	T/C	0.68	Increases	2.56x10^-12^	No (rs11014171) (18,751,201) (r^2^=0.61)	C/T	0.85	Decreases	NA		1.21	(0.73-2.02)	0.46
10	*C10orf107*	rs4590817 (63,137,559)	G/C	0.84	Increases	3.97x10^-12^	No (rs12246717) (63,129,189) (r^2^=0.62)	T/G	0.71	Decreases	NA		1.04	(0.76-1.42)	0.81
10	*PLCE1*	rs932764 (95,885,930)	G/A	0.44	Increases	7.10x10^-16^	No (rs2901761) (95,885,117) (r^2^=0.60)	A/G	0.17	Increases	0.01		1.01	(0.76-1.35)	0.94
10	*CYP17A1-NT5C2*	rs11191548 (104,836,168)	T/C	0.91	Increases	6.90x10^-26^	Yes	T/C	0.97	Increases	0.32		0.39	(0.13-1.13)	0.08
11	*ADM*	rs7129220 (10,307,114)	G/A	0.89	Decreases	2.97x10^-12^	No (rs7929332) (10,180,933) (r^2^=0.72)	T/C	0.91	Decreases	0.28		1.57	(0.90-2.76)	0.11
11	*PLEKHA7*	rs381815 (16,858,844)	T/C	0.26	Increases	5.27x10^-11^	Yes	T/C	0.17	Increases	0.46		0.64	(0.42-0.99)	0.04
11	*FLJ32810-TMEM133*	rs633185 (100,098,748)	G/C	0.28	Decreases	1.21x10^-17^	No (rs6590810) (100,083,885) (r^2^=0.63)	A/G	0.27	Decreases	0.05		0.92	(0.66-1.27)	0.59
12	*ATP2B1*	rs17249754 (88,584,717)	G/A	0.84	Increases	1.82x10^-18^	No (rs6538195) (88,586,507) (r^2^=1)	G/A	0.89	Decreases	NA		0.92	(0.61-1.41)	0.72
12	*SH2B3*	rs3184504 (110,368,991)	T/C	0.48	Increases	3.83x10^-18^	Yes	T/C	0.07	Increases	0.43		0.7	(0.46-1.06)	0.1
12	*TBX5-TBX3*	rs10850411 (113,872,179)	T/C	0.7	Increases	5.38x10^-8^	Yes	T/C	0.63	Increases	0.38		0.79	(0.54-1.15)	0.22
15	*CYP1A1-ULK3*	rs1378942 (72,864,420)	C/A	0.35	Increases	5.69x10^-23^	Yes	C/A	0.9	Decreases	NA		1.36	(0.80-2.30)	0.26
15	*FURIN-FÈS*	rs2521501 (89,238,392)	T/A	0.31	Increases	5.20x10^-19^	No (rs1029420) (89,242,090) (r^2^=0.70)	C/T	0.29	Decreases	NA		1.21	(0.89-1.64)	0.22
17	*GOSR2*	rs17608766 (42,368,270)	T/C	0.86	Decreases	1.13x10^-10^	Yes	T/C	0.98	Increases	NA		2.35	(1.15-4.81)	0.02
17	*ZNF652*	rs12940887 (44,757,806)	T/C	0.38	Increases	1.79x10^-10^	No (rs17637472) (44,816,432) (r^2^=0.62)	A/G	0.06	Increases	0.03		0.79	(0.49-1.27)	0.33
20	*JAG1*	rs1327235 (10,917,030)	G/A	0.46	Increases	1.87x10^-8^	No (rs1887320) (10,913,998) (r^2^=1.0)	G/A	0.48	Increases	0.08		0.98	(0.79-1.20)	0.82
20	*GNAS-EDN3*	rs6015450 (57,184,512)	G/A	0.12	Increases	3.87x10^-23^	No(rs6026742) (57,174,000) (r^2^=0.85)	A/G	0.22	Increases	0.06		1.22	(0.89-1.69)	0.22

Genomic positions are in reference to NCBI build 36.3

Chr: chromosome; CA: coded allele; OA: other allele; CAF: coded allele frequency; OR: odds ratio; CI: confidence interval

In the absence of the genotype data for the reported index SNP, best proxy was selected using HapMap phase III (release 2, ASW panel) and 1000 Genomes project (Pilot 1, YRI panel ) with cutoff r^2^ ≥ 0.6

† Observed effects are based on inverse variance weighted meta-analysis*P-values are based on one-tailed significanceNA indicates opposite direction of effect between ICBP and SCD cohorts

‡ Odds ratio based on the multivariate logistic regression adjusted for age, sex and 1st principal component

### Association of SBP reported loci with SCI

Given that higher SBP is associated with increased risk of an SCI in SCD patients, we determined whether any of the SNPs (or their proxies) associated with SBP in ICBP study was associated with SCI. Three SNPs were nominally associated with SCI (Table **4**), with only the *CACNB2* (*5’*) locus (rs2357790) consistent between the SBP (P-value=0.05) and SCI (P-value=0.04) analyses. None of these SNPs was significant after Bonferroni correction. Further, to estimate the aggregated effect of these SNPs on increased risk of SCI, we constructed the genetic risk score for SCI (weighted according to effect sizes of published SBP SNPs) and no significant association was observed (P-value= 0.95).

## Discussion

In recent years, genome-wide scans demonstrated a successful means of identifying novel common genetic variants that contribute to susceptibility to complex diseases, including blood pressure [21-23,39,40]. Here, we present the results from a meta-analysis of SBP from two SCD cohorts comprised of 1,617 SCD patients, all with African American ancestry. No associations were genome-wide significant; however we observed suggestive association at rs7952106, a 5’ upstream SNP to *DRD2* gene on chromosome 11, which showed consistent association evidence in both the studied cohorts. Further, in a gene-based test, a suggestive signal of non-coding RNA (*MIR4301*) and the prediction of *MIR4301* binding to *DRD2* as a potential target, suggests the plausible involvement of *DRD2* region in the regulation of SBP in SCD cohorts.

Previously, several studies have shown that dopamine synthesis in the kidney has an important role in the regulation of fluid and electrolyte balance and systemic blood pressure [41-43]. Dopamine exerts its actions via 2 families of G-protein-coupled receptors D1-like receptors (DRD1 and DRD5) and D2-like receptors (DRD2, DRD3, and DRD4). Later, several lines of evidence also showed that an intact dopaminergic system is necessary to maintain normal blood pressure and that genetic hypertension is associated with alterations in dopamine production and receptor function [41-44]. Deletion of any dopamine receptor in mice results in increased blood pressure by mechanisms that are receptor dependent. In particular, mice lacking the DRD2 gene (DRD2-/-

) have reactive oxygen species (ROS)-dependent hypertension [44]. In addition, the *DRD2* polymorphisms were also reported with decreased *DRD2* expression [45] and shown to affect *DRD2* mRNA stability and synthesis of the receptor [46]. These studies suggest that the *DRD2* locus is plausibly involved in the regulation of SBP. Our results support these findings and the suggestive association from the *DRD2* region SNPs may represents a true signal associated with SBP in SCD patients.

Recently, several large and well powered studies from European ancestry populations have identified 29 genomic loci associated with blood pressure [21-23]. We sought to validate these reported loci in the setting of SCD in populations of African American ancestry, and to further test whether any of these loci were involved in SCI. Our study reports that the derived genetic risk scores for SBP is significantly associated with SCD children of African American ancestry. The significant association of the aggregated genetic risk scores with SBP in our study highlights the importance of these loci in the modulation of SBP in the studied SCD cohort.

In SCD patients, neurovascular complications are common and largely due to tissue ischemia and infarctions [13,47]. SCI is a major cause of neurologic morbidity in SCD children with unclear genetic susceptibility [47,48]. Given that hypertension is a known risk factor for stroke and more recently, SBP has been reported associated with risk for SCI [13,49], identifying genetic variants associated with SBP in SCD patients may also lead to the identification of genes associated with SCI. Although we confirm the association of 4 loci with SBP, only the *CACNB2* (*5’*) locus showed the consistent nominal significance for both SBP and SCI (Table **4**).

A few limitations to the current study need to be acknowledged. First, although we observed a significant association of the aggregated genetic risk score with SBP, we failed to reproduce the significance of any individual SBP associated SNPs after multi-test correction and this may be due to the limited sample size of our study. As shown in Figure **S3**, our study (n=1,617) was also under-powered to identify novel genome-wide significant variants. Secondly, biological differences that exist between ethnic groups and complex interaction of age with different genetic alteration may also have negatively impacted our ability to identify significant loci. Also, the admixed nature of the African ancestry population may lead to differences in local LD patterns. Since we are not likely to be genotyping the functional variant, changes in LD patterns between Europeans and the studied African American populations can change the nature of the observed associations. Thirdly, the ability to detect genetic determinants associated with any trait of interest largely depends on the quality and reliability of the data. In our study, it is noteworthy to highlight that we have used the longitudinal SBP measurements from the CSSCD cohort, as oppose to the single time point data available from the SIT Trial. In addition, CSSCD SBP measurements were taken after following the procedure described in the study manual of operations, and hence provides more certainty of less variability, whereas, no uniform guidelines were adopted in the SIT Trial. At last, it has also been known for over a decade that blood pressure is an age-dependent process [50,51]. The ICBP study was performed in adult individuals (38-72 yrs), whereas in our study, SCD patients are restricted to age < 15 years; therefore, it is possible that the lack of association for other loci may be due to an age-dependent genetic effect.

In summary, our results not only suggest the importance of *DRD2* genetic variation in the modulation of SBP, but also extend the genetic significance of the 4 previously published loci in SBP in an African American population. Further, our study also identifies a significant association for the genetic risk score with SBP, suggesting the aggregated importance of previously reported SNPs in the modulation of SBP in the setting of SCD. This study provides new insight in SBP regulation in an admixed African American ancestry cohort, more specifically in children with SCD, and highlights the overlap in genetic signals between African American populations and European ancestry populations.

## Supporting Information

Figure S1 **A quantile-quantile (Q-Q) plot showing the distribution of observed χ^2^ statistics of analyzed SNPs in 1,617 SCD individuals (SIT Trial, n=925; CSSCD, n=692).** The black diagonal line indicates expected results under the null hypothesis.
(TIFF)

Figure S2 **Comparison of the effect size of variants from the reported regions associated with SBP in the ICBP meta-analysis and SCD cohort.**
(TIFF)

Figure S3 **Power calculation to detect genome-wide significant SNPs (minor allele frequency > 0.05) for systolic blood pressure in the combined SIT Trial and CSSCD cohort, utilizing 1617 SCD samples.** The authors thank the staff, clinicians and patients for their participation in the Silent Infarct Transfusion (SIT) Trial study. Genotyping services for the SIT Trial were provided by the Center for Inherited Disease Research (CIDR) at Johns Hopkins University and the Division of Blood Disorders at the Centers for Disease Control and Prevention (CDC). The findings and conclusions in this report are those of the authors and do not necessarily represent the official position of the Centers for Disease Control and Prevention.
(TIFF)
