# Supplementary figures and images for: Genome-Wide Meta-Analysis of Systolic Blood Pressure in Children with Sickle Cell Disease
Source: PLoS One. 2013 Sep 13;8(9):e74193. doi: 10.1371/journal.pone.0074193 (PMC3772989; doi:10.1371/journal.pone.0074193)

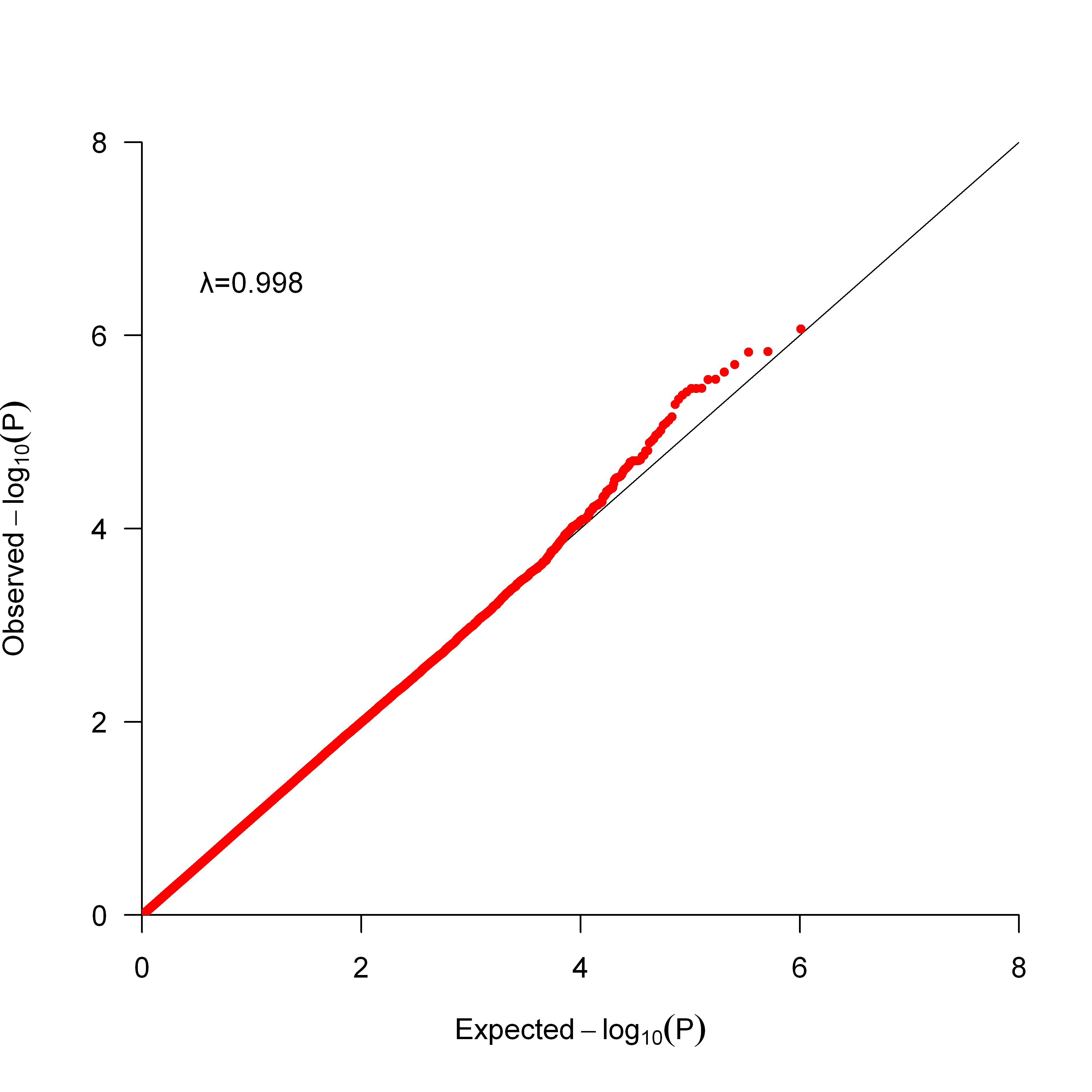

Supplement: Figure S1 — A quantile-quantile (Q-Q) plot showing the distribution of observed χ2 statistics of analyzed SNPs in 1,617 SCD individuals (SIT Trial, n=925; CSSCD, n=692). The black diagonal line indicates expected results under the null hypothesis. (TIFF) [file pone.0074193.s001.tiff]

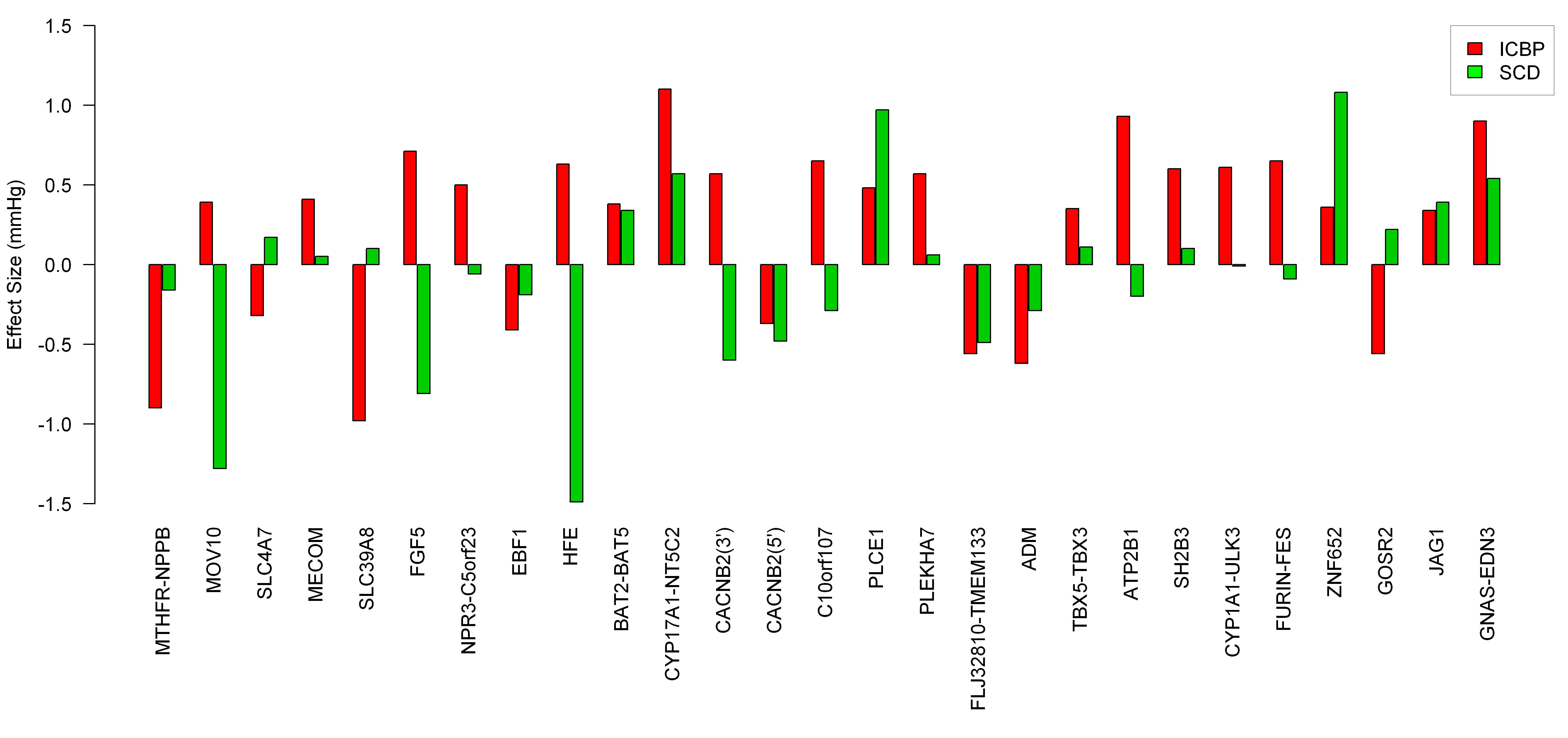

Supplement: Figure S2 — Comparison of the effect size of variants from the reported regions associated with SBP in the ICBP meta-analysis and SCD cohort. (TIFF) [file pone.0074193.s002.tiff]

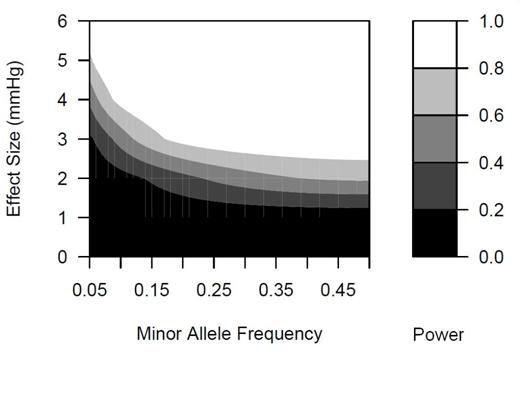

Supplement: Figure S3 — Power calculation to detect genome-wide significant SNPs (minor allele frequency > 0.05) for systolic blood pressure in the combined SIT Trial and CSSCD cohort, utilizing 1617 SCD samples. The authors thank the staff, clinicians and patients for their participation in the Silent Infarct Transfusion (SIT) Trial study. Genotyping services for the SIT Trial were provided by the Center for Inherited Disease Research (CIDR) at Johns Hopkins University and the Division of Blood Disorders at the Centers for Disease Control and Prevention (CDC). The findings and conclusions in this report are those of the authors and do not necessarily represent the official position of the Centers for Disease Control and Prevention. (TIFF) [file pone.0074193.s003.tiff]
